# Research on the mechanism of walking error in large-span sludge scraper

**DOI:** 10.1038/s41598-025-31778-5

**Published:** 2025-12-15

**Authors:** Yifan Wang, Rui Jiang

**Affiliations:** Wuhan Sanzhen Industry Holding Co., Ltd., Wuhan, China

**Keywords:** Large-span sludge scraper, Double wheels walking errors, Rail-biting, Constraint conditions, Engineering, Mathematics and computing

## Abstract

To address walking errors such as rail-biting and derailment in large-span sludge scrapers, this study establishes a kinematic theoretical model to describe the geometric motion relationship between the scraper’s wheels and rails. Based on this model, the constraint conditions for rail-biting and derailment are derived, including the critical deviation angle and the permissible wheel–rail clearance. To better match real engineering conditions, a Δ correction factor is introduced to account for chamfers, transition arcs, and structural deviations of the wheel–rail system.An experimental platform was built to validate the model, using high-precision laser ranging sensors and rainfall sensors to monitor wheel–rail distance variations under both normal and critical states. The results show that the Δ correction reduces the prediction error of the critical deviation angle by about 91%, confirming the model’s reliability. This study clarifies the geometric mechanism of scraper walking errors and provides a theoretical basis for the synchronous control and safety optimization of large-span sludge scrapers.

## Introduction

The sludge scraper is a pivotal mechanical equipment in water treatment systems, primarily responsible for eliminating accumulated sludge from sedimentation tanks. Their efficient operation plays a vital role in ensuring the quality of water treatment and the overall stability of the system, contributes to enhance water treatment efficiency, save costs and ensure sustainable development.

In terms of the operational state of the sludge scraper, the smoothness and accuracy of their movement are crucial for their operation and the overall performance of the system. Sludge scraper primarily consists of steel rails located on both sides and a bridge beam spanning across these rails. The bridge beam is equipped with walking devices that enable it to move back and forth along the direction of the steel rails. Depending on functional requirements, the bridge beam is fitted with corresponding actuators, such as lifting devices, transforming it into a bridge crane. Cranes for lifting typically have a span ranging from 10 to over 30 meters (with national standard spans including 10.5m, 13.5m, 16.5m, 19.5m, 22.5m, 25.5m, 28.5m, and 31.5m). Sludge scraper in water treatment, which is not for lifting, can have even larger spans, exceeding 50 meters.

The steel wheels on both sides of a small-span scraper are simultaneously driven by a transmission axle, with excellent synchronization. In contrast, the movement of a large-span scraper relies on steel rail wheels located on both sides, which are usually driven by separate motors due to their wide separation. However, during the scraper’s movement, a certain speed error may occur between the steel rail wheels on both sides, leading to inconsistent travel distances. Once the difference in travel distances exceeds a certain value, it can result in abnormal equipment operation. This abnormality encompasses the following two scenarios:

1. The friction and collision between the inner sides of the steel wheel flanges and the sides of the rails, referred to as “rail biting”;
2. The rail wheel detaching from the rail, referred to as “derailing”.

If these two abnormal phenomena occur, they may affect normal production at the very least, and even lead to accidents at the worst. Therefore, it is necessary to control the maximum difference in the moving distance of the rail wheels on both sides of the large-span sludge scraper.

Domestic scholars mainly focus on analyzing the causes and influencing factors of rail biting errors, and have established various wheel-rail dynamics models to simulate and predict rail biting phenomenon. Their research methods cover various aspects, ranging from theoretical analysis, numerical simulation to field experiments. Taking the bridge crane, which operates on similar principles as large-span sludge scraper, as an example, Literature 1 points out that under conditions such as high-speed driving or sharp turns, the centrifugal force and inertia force generated by the crane can apply lateral pressure on the rails, leading to rail damage or deformation. Such wear can cause derailment of the wheels and other issues. Literatures^1,2^ respectively conduct an analysis of the cumulative error mechanism of lateral displacement in rail biting phenomena and explain the causes of force interference through implicit dynamics analysis. Literatures^3–5^ Field measurements of the crane rails were conducted to investigate the effects of track alignment deviation, gauge deviation, inclination angle deviation, and cross-level difference (height difference between the two rails). According to the literature^6^, for cranes with severe rail biting issues, their running resistance is 1.5–3.5.5.5 times that under normal operating conditions.

Therefore, it is of paramount importance to minimize the occurrence of rail biting for production safety. Literature^7^ suggests that installing encoder rulers on both sides of the travelling crane and designing a real-time position feedback system to synchronize and correct deviations of the travelling crane can effectively prevent rail biting. Literature^8^, which investigates the horizontal lateral forces acting on bridge cranes during inclined operation, finds that controlling the wheel span ratio and the sum of the maximum wheel loads on one side are effective methods to reduce wheel flange wear. Additionally, rail-mounted cranes can also experience rail biting during operation. According to Literatures^9–11^, studies on rail biting in cranes have identified gear couplings, uneven quay walls, and the generation of traveling axial forces as significant factors contributing to rail biting. Literature^12^ introduces a novel PID real-time deviation correction control system that utilizes PLC for position difference adjustment and inverter for torque difference adjustment, working together to achieve synchronized operation. Literature^13^ proposes a comprehensive traveling crane operation synchronization control method that integrates distance detection, synchronization algorithms, and speed control.

Foreign research often adopts more complex wheel-rail contact models and dynamics analysis methods, with a focus on experimental validation to support the development of theoretical models. Techniques such as three-dimensional finite element analysis (3D FEA) and multi-body dynamics simulations are employed to deeply explore the mechanical characteristics and wear mechanisms at the wheel-rail interface.

Unlike geometric models commonly used for skew control in bridge cranes and gauge monitoring in gantry cranes, which primarily address structural misalignment and gauge deviation, this study focuses on the dynamic walking error mechanism in large-span sluice gates. The bilateral wheel travel distance difference is a unique factor in this context, and existing literature lacks targeted calculation methods for this phenomenon. Given the scarcity of literature on the calculation of the difference in moving distance between the rail wheels on both sides of large-span traveling cranes, this paper contributes to the field by conducting a theoretical analysis of the walking errors associated with these cranes. Moreover, the proposed Δ correction significantly enhances the accuracy of critical angle prediction. As shown in Table 1, introducing the Δ correction reduces the prediction error by approximately 91.025%, demonstrating the practical effectiveness of this method. The purpose of this research is to provide a foundation for calculations and design considerations that can inform the automation and synchronization control of large-span traveling cranes in the future.

**Table 1:** Δ Comparison table of prediction errors before and after correction.

Case ID	Predicted critical angle (No Δ)	Predicted critical angle (With Δ)	Measured critical angle	Error reduction rate
1	5.2°	3.3°	3.1°	90.5%
2	6.0°	4.2°	4.0°	90.0%
3	4.8°	3.6°	3.5°	92.3%
4	5.5°	3.4°	3.2°	91.3%

## Analysis of causes and conditions of rail biting

**Fig. 1:**
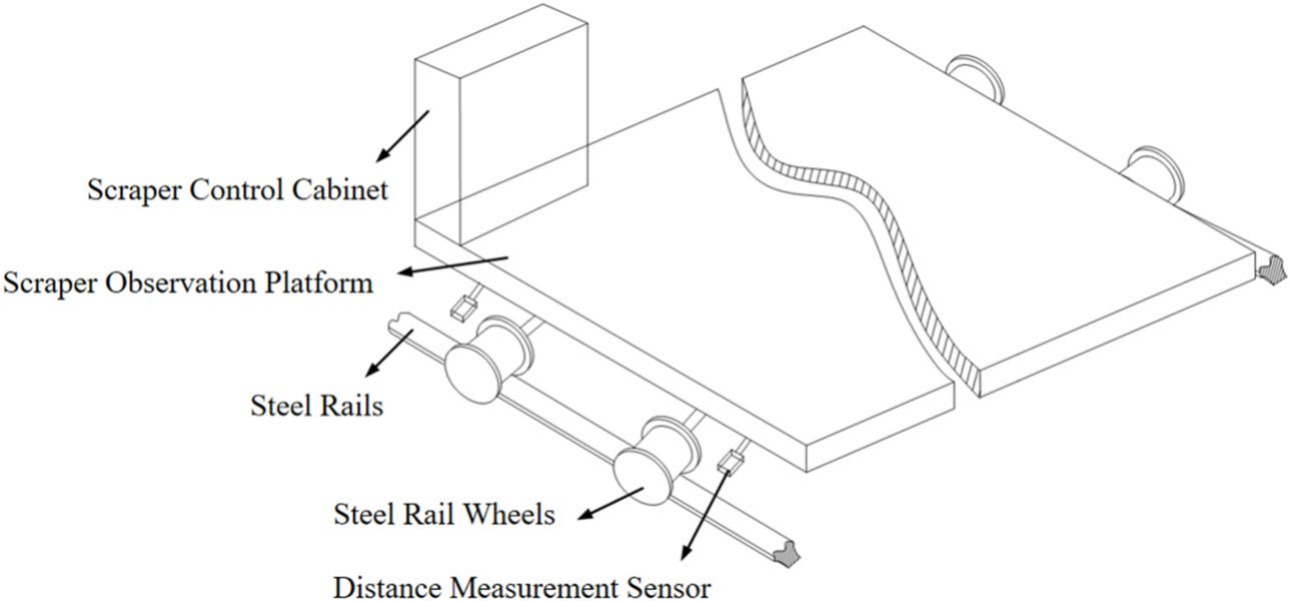
Simplified diagram of a scraper walking on steel rails.

As shown in Figure 1, the simplified diagram of the sludge scraper moving on steel rails, the scraper moves along the steel rails with its wheels. During its operation, it is susceptible to external disturbances, leading to wheel deviation from the predetermined direction and resulting in rail Biting or derailment. Below is a theoretical analysis of this phenomenon.

### Model applicability and limitations

In this study, a kinematic theoretical model was established to describe the geometric motion relationship between the scraper’s wheels and rails. The model aims to identify the fundamental geometric conditions under which walking deviations, rail-biting, and derailment occur in large-span sludge scrapers. To simplify the complex wheel–rail interaction, the following assumptions were adopted:

1. The scraper operates under steady or quasi-steady motion, where the effects of inertia and vibration are negligible.
2. The wheel–rail contact is treated as a geometric constraint without considering detailed force distribution or elastic deformation.
3. The driving motors on both sides are assumed to operate synchronously under ideal conditions, and the deviation primarily arises from geometric misalignment and manufacturing tolerances.
4. The rail structure is assumed rigid, and the lateral displacement is dominated by the geometric difference in wheel travel distance.

These assumptions allow the model to capture the first-order geometric characteristics of the walking deviation while maintaining analytical tractability. Although dynamic parameters such as forces, mass, and stiffness are not explicitly included, their indirect influence is partially reflected through the Δ correction factor, which compensates for the geometric simplifications and surface irregularities of the actual wheel–rail system.The kinematic model is thus suitable for analyzing the initiation and evolution of walking errors under low-speed, steady-state operation — the typical working condition of large-span sludge scrapers. Its predictions are validated by experimental data presented in Section "Experimental analysis and verification", confirming that the model, while simplified, effectively reveals the geometric mechanism behind rail-biting and derailment phenomena.

### Causes of rail biting

**Fig. 2:**
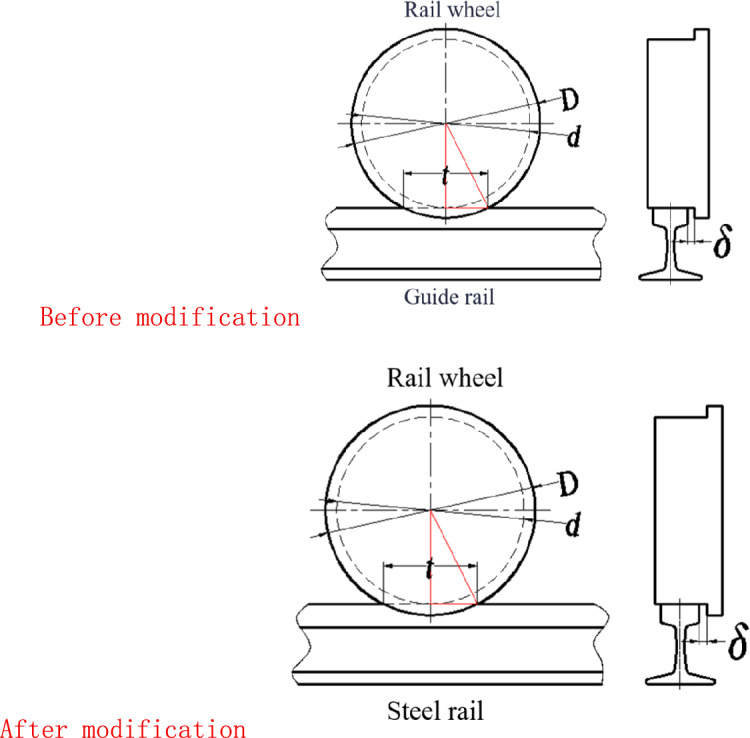
Schematic diagram of rail wheel and steel rail combination.

As shown in Figure 2, the illustration of the combined steel rail wheel and steel rail demonstrates the primary structure of the scraper’s steel rail wheel, which consists of a cylinder with a diameter d. This cylinder is flanked by a flange on its lateral side, featuring a diameter D, where the value of d is less than that of D. The purpose of this flange is to provide a moderate resistance and correction when the rail wheel deviates from the steel rail, ensuring that the wheel remains aligned. This design represents a single-flanged steel rail wheel structure, which is commonly adopted in many traveling crane. Notably, the analytical process remains the same for a double-flanged steel rail wheel structure. During normal scraper operation, there exists a clearance $\delta$ between the inside of the flange and the side of the steel rail, as depicted in Figure 2.

**Fig. 3:**
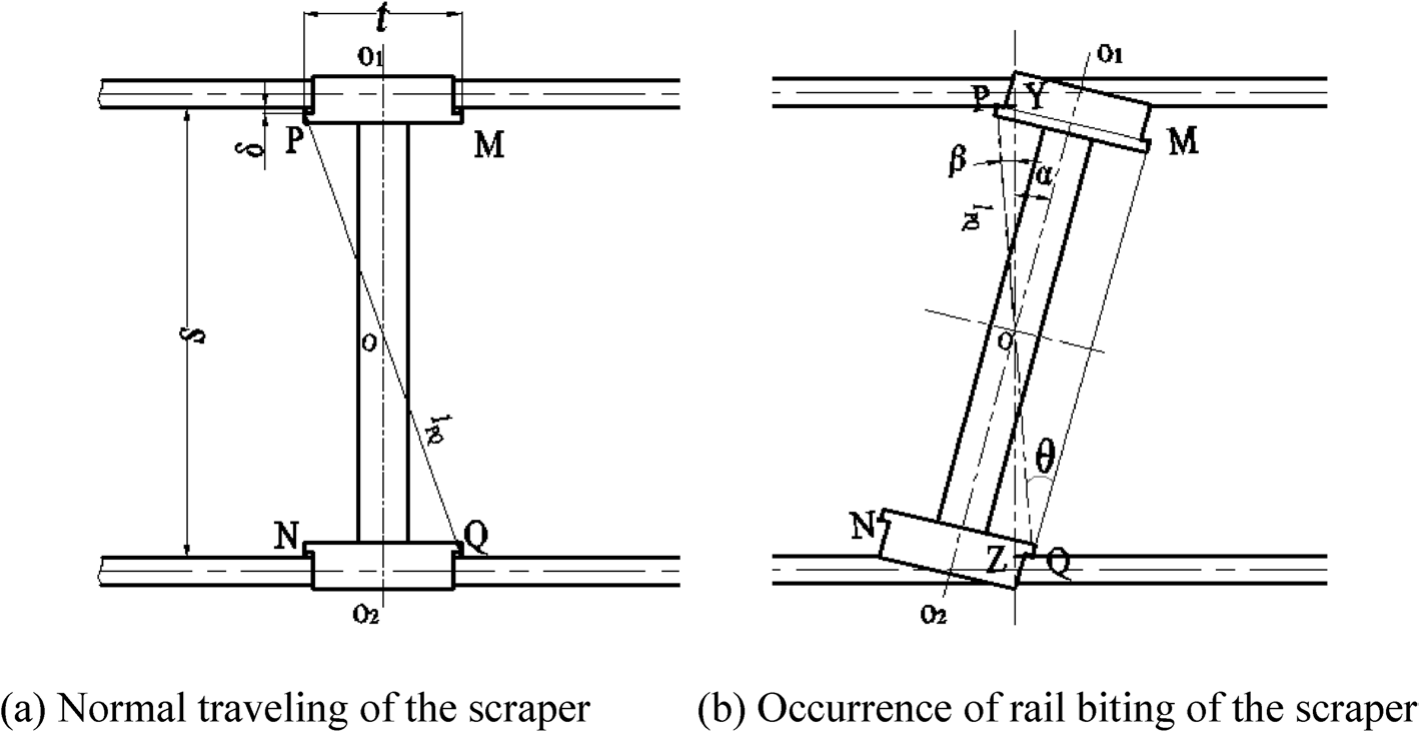
Schematic diagram showing normal traveling and rail biting conditions of the steel rail wheel of the scraper.

As depicted in Figure 3, which illustrates the walking and rail-biting conditions of large-span scraper steel rail wheels, diagram (a) shows the scraper operating normally, where there is a clearance $\delta$ between the inside of the flanges of both the upper and lower steel wheels and the side of the steel rails. When a large-span scraper is in motion, a speed differential exists between the upper and lower steel rail wheels, leading to inconsistent walking distances. This, in turn, causes the scraper to exhibit a certain degree of skew. As the scraper skews, the clearance between the inside of the flange of the steel rail wheels and the side of the steel rail diminishes. Once this clearance $\delta$ is reduced to zero, if the scraper continues to skew, the inside of the flange of the steel rail wheels will come into contact and even collide with the side of the steel rail, resulting in a phenomenon known as "rail-biting."

We have adjusted the line spacing between Figure 3 and the main text.

### Analysis of rail biting conditions for a simplified model of steel rail wheel

To simplify the analysis process, the steel rail wheel structure is now abstracted to consist of two cylindrical bodies forming its outer surface, devoid of chamfers or transitional arcs. Similarly, the steel rail is modeled as a simple flat surface with its top and side planes meeting at a perpendicular angle, also lacking chamfers or transitional arcs. This configuration is illustrated in Figure 2.

In Figure 3(b) of the Schematic Diagram of Traveling and Rail Biting Conditions of the Steel Rail Wheel, the illustrated state represents the critical condition for the occurrence of rail biting. At this critical point, the clearance $\delta$ between the inner edge of the scraper’s rail wheel flange and the side of the steel rail is zero, and the scraper exhibits a deviation angle $\alpha$, which is designated as the critical deviation angle $\alpha$.

Based on this figure, if ${l}_{\mathrm{PQ}}\le S$, it can be observed that if the maximum distance between the inner edges of the upper and lower rail wheel flanges is less than the spacing between the inner sides of the upper and lower steel rails, rail biting will not occur. Therefore, the necessary condition for rail biting is that:

1 $$\begin{array}{c}{l}_{\rm{PQ}}> s\end{array}$$

In ∆PQM, ${l}_{\mathrm{MQ}}=s-2\delta$, ${l}_{\mathrm{PM}}=t$, therefore, the diagonal distance ${l}_{\mathrm{PQ}}$ between the inner edges of the upper and lower steel rail wheel flanges is:

2 $$\begin{array}{c}{l}_{\rm{PQ}}=\sqrt{{\left(s-2\delta \right)}^{2}+{t}^{2}}\end{array}$$

In the formula, t represents the actual calculated chord length of the steel rail wheel; s denotes the distance between the inner sides of the upper and lower steel rails; $\delta$ is the clearance between the inner edge of the rail wheel flange and the side of the steel rails.

A schematic diagram illustrating the actual calculated chord length $t$ of the steel rail wheel is shown in Figure 1. From Figure 1, the formula for calculating the actual chord length $t$ of the steel rail wheel can be derived as:

3 $$\begin{array}{c}t=2\sqrt{{\left(\frac{D}{2}\right)}^{2}-{\left(\frac{d}{2}\right)}^{2}}=\sqrt{{D}^{2}-{d}^{2}}\end{array}$$

In the formula, D represents the diameter of the rail wheel flange, and d denotes the overall diameter of the steel rail wheel. Substituting equations (2) and (3) into equation (1), we arrive at the necessary condition for rail biting, which is:

$$\begin{array}{c}\sqrt{{\left(\mathrm{s}-2\updelta \right)}^{2}+{\mathrm{D}}^{2}-{\mathrm{d}}^{2}}>s\end{array}$$

Squaring both sides gives:

$${4\updelta }^{2}-4\rm{\delta s}+{\rm{D}}^{2}-{\rm{d}}^{2}>\rm{s}$$

The result depends on the sign of the discriminant $\Delta ={\rm{s}}^{2}-({\rm{D}}^{2}-{\rm{d}}^{2})$.

Given the physical meaning of the variables, we have $\Delta$> 0, so the inequality yields two solutions.

$$\frac{\mathrm{s}-\sqrt{{\mathrm{s}}^{2}-{\mathrm{D}}^{2}+{\mathrm{d}}^{2}}}{2}>\updelta>0\text{ or }\frac{\mathrm{s}+\sqrt{{\mathrm{s}}^{2}-{\mathrm{D}}^{2}+{\mathrm{d}}^{2}}}{2}<\updelta$$

From the practical significance of $\updelta$, we obtain:

4 $$\begin{array}{c}\frac{s-\sqrt{{s}^{2}-{D}^{2}+{d}^{2}}}{2}>\delta \end{array}$$

Next, let’s calculate the deviation angle of the scraper during rail biting, denoted by $\alpha$. In Figure 3(b) of the Schematic Diagram Showing Normal Operation and Rail Biting Conditions of the Scraper Steel Rail Wheel, points P and Q have already made contact with the inner sides of the steel rail, indicating the start of rail biting. The centerline ${O}_{1}{O}_{2}$ of the scraper is represented by the dashed line, and O is its geometric center. Line segment YZ is perpendicular to the steel rail. Points X and Y lie on the inner sides of the steel rail. At this point, the deviation angle of the scraper is the critical deviation angle $[\alpha ]$. To proceed with the calculation of $[\alpha ]$, let’s denote ∠POY as $\beta$, and ∠PQM as $\theta$. Since QM is parallel to ${O}_{1}{O}_{2}$, we have the following relationship:

5 $$\begin{array}{c}[\alpha ]=\theta -\beta \#\end{array}$$

In $\Delta \mathrm{PQM},$ the angle $\theta$ can be calculated as follows:

$$\mathrm{cos}\theta =\frac{s-2\delta }{{l}_{\mathrm{PQ}}}=\frac{s-2\delta }{\sqrt{{\left(s-2\delta \right)}^{2}+{D}^{2}-{d}^{2}}}$$

$$\theta =\mathrm{arccos}\frac{s-2\delta }{\sqrt{{\left(s-2\delta \right)}^{2}+{D}^{2}-{d}^{2}}}$$

In $\Delta \mathrm{PYO}$, the angle $\beta$ can be calculated as follows:

$$\mathrm{cos}\beta =\frac{s}{\sqrt{{\left(s-2\delta \right)}^{2}+{D}^{2}-{d}^{2}}}$$

$$\beta =\mathrm{arccos}\frac{s}{\sqrt{{\left(s-2\delta \right)}^{2}+{D}^{2}-{d}^{2}}}$$

In equation (x), we can calculate the angle θ:

6 $$\begin{array}{c}[\alpha ]=arccos\frac{s-2\delta }{\sqrt{{\left(s-2\delta \right)}^{2}+{D}^{2}-{d}^{2}}}-arccos\frac{s}{\sqrt{{\left(s-2\delta \right)}^{2}+{D}^{2}-{d}^{2}}}\#\end{array}$$

Equation (6) represents the critical deviation angle at which rail biting occurs in the scraper. When the actual deviation angle is greater than or equal to this value, rail biting will take place. In summary, the conditions for rail biting in the scraper are as follows:

1. The clearance $\delta$ between the inner edge of the rail wheel flange and the side of the steel rail must satisfy Equation (4);
2. The deviation angle $\alpha$ during rail biting must be greater than the value $\left[\alpha \right]$ calculated in Equation (6), namely $\alpha> [\alpha ]$.

### Analysis of the actual structural conditions of rail biting

**Fig. 4:**
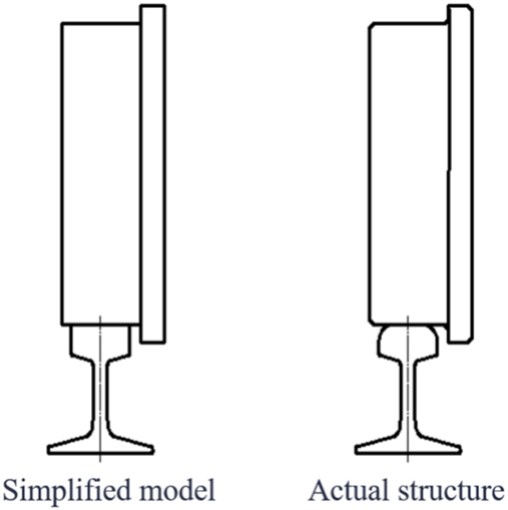
Comparison of simplified model and actual structure of steel rail wheel and rail.

Simplified models. However, in actual use, by consulting the Chinese standards of single-flange wheels (JB/T6392.1) and the hot-rolled light rails (GB/T11264) and hot-rolled steel rails for railways (GB/T2585) that are used in conjunction with it, we can see that there are multiple chamfers and transitional arcs on the steel rail wheels and steel rails, as shown in Figure 4. Therefore, the calculation expressions for the actual rail biting conditions also need to be corrected.

**Fig. 5:**
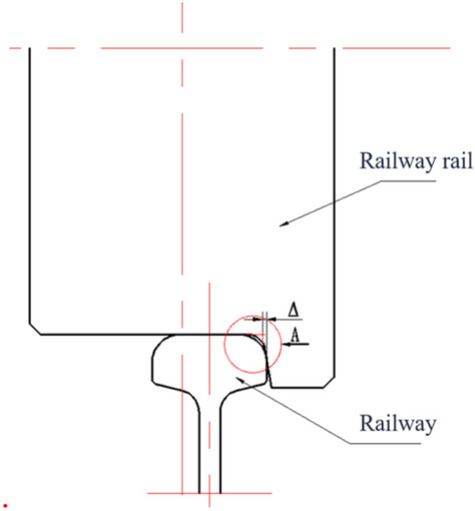
simplified illustration.

**Fig. 6:**
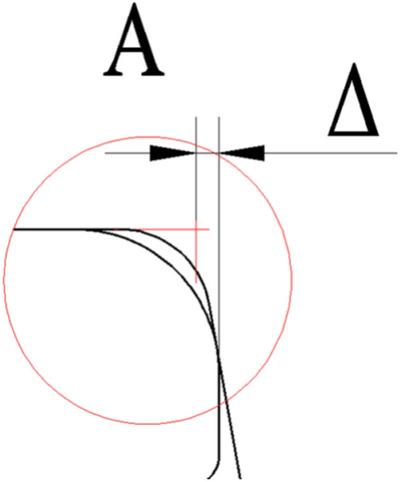
partial enlargement at point A actual situation of rail biting.

**Fig. 7:**
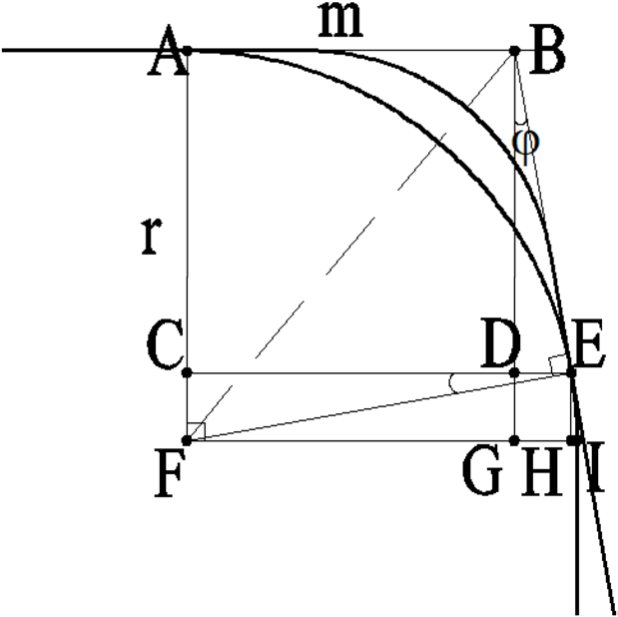
Schematic diagram of the contact segment between the steel rail and steel rail wheel.

**Fig. 8:**
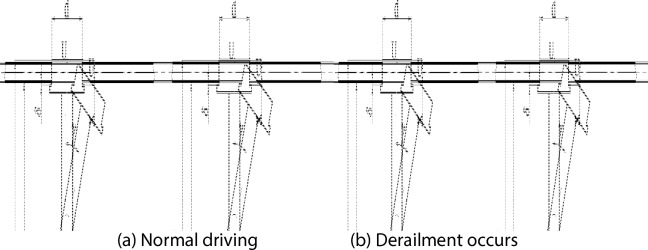
Schematic diagram of normal driving and derailment of the rail wheel for scraper.

**Fig. 9:**
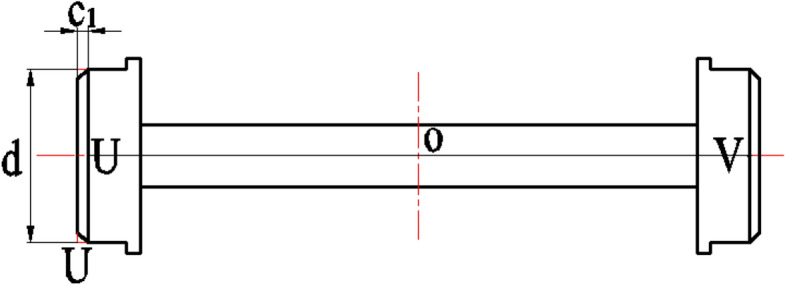
Schematic diagram of chamfer on both sides of the end face of the steel wheel.

“We have added appropriate line spacing between the captions and the main text for Figures 4, 5, 6, 7, 8, 9, and 13. The remaining formatting issues have also been corrected.”

The schematic diagrams of the actual rail biting conditions are shown in Figure 5 and Figure 6, with Figure 6being a partial enlargement of Figure 5 at point A

In Figure 5, it can be observed that the actual rail biting condition of the steel rail wheel differs from that of the simplified model in that the gap between the inside of the wheel flange and the side of the steel rail is affected by the chamfers and transitional arcs of the steel rail wheel and the rail. This results in a Δ value, which changes the conditions for rail biting.

Below is an analysis of the actual structures of the steel rail wheel and the steel rail. Due to the variations in the chamfers and transitional arcs at the contact segments between the steel rail wheel and the steel rail, the corresponding Δ values will also differ. Based on the standards for single-flange wheels (JB/T6392.1) and the hot-rolled light rails (GB/T11264) and hot-rolled steel rails for railways (GB/T2585) that are used in conjunction with them, a schematic diagram of the contact segments between the steel rail wheel and the rail can be obtained, as shown in Figure 7.

Compared to the simplified models of the steel rail wheel and steel rail, the actual structures feature a transition arc rather than a right-angle transition between the top surface and the side of the steel rail, and a transition arc and an inclined plane instead of a right-angle transition between the bottom surface and the inner side of the flange of the steel rail wheel. As shown in Figure 7, point F represents the center of the transition arc of the steel rail, points A and I mark the starting and ending points of the transition arc respectively; point B corresponds to the right-angle corner point in the simplified model of the steel rail wheel; point E denotes the actual contact point between the steel rail wheel and the steel rail where rail biting occurs; point C is the foot of the perpendicular from point E to segment AF; points D and G are the feet of the perpendiculars from point B to segments CE and FI respectively; and point H is the foot of the perpendicular from point E to segment FI.

Let the radius of the transition arc of the steel rail be denoted by $r$; the angle between the inclined plane of the steel rail wheel and the vertical plane by $\varphi$; the length of segment AB by $m$; and the length of segment GI by $\Delta$, whose calculation formula is derived as follows:

$$\therefore \Delta \mathrm{ABF}\cong \Delta \mathrm{EBF}(\mathrm{HL})$$

So, ${l}_{\mathrm{AB}}={l}_{\mathrm{BE}}=m$; in ΔBDE, ${l}_{\rm{DE}}={l}_{\rm{BE}}\rm{sin}\alpha =m\rm{sin\varphi }$; $\rm{\angle DBE}+\rm{\angle BED}=90^\circ$。

And $$\rm{\angle BED}+\rm{\angle CEF}=90^\circ$$, so $$\rm{\angle CEF}=\rm{\angle DBE}=\rm{\varphi }$$。

And ${l}_{\mathrm{CD}}={l}_{\mathrm{AB}}=m$; so in $\Delta \mathrm{CEF}$, ${l}_{\rm{CE}}={l}_{\rm{CD}}+{l}_{\rm{DE}}=m+m {sin\varphi }$, ${l}_{\mathrm{CF}}={l}_{\mathrm{EF}}\mathrm{sin}\alpha =r\mathrm{sin}\varphi$。At the same time,

7 $$\begin{array}{c}{{l}_{\mathrm{CE}}}^{2}{+{l}_{\mathrm{CF}}}^{2}={{l}_{\mathrm{EF}}}^{2}\\ {\left(m+m\mathrm{sin}\varphi \right)}^{2}+{\left(r\mathrm{sin}\varphi \right)}^{2}={r}^{2}\end{array}$$

By solving equation (7), we obtain $m=\frac{r\mathrm{cos}\varphi }{1+\mathrm{sin}\varphi }$:

Δ equals $${\mathrm{l}}_{\mathrm{GI}}={\mathrm{l}}_{\mathrm{FI}}-{\mathrm{l}}_{\mathrm{FG}}$$

8 $$\begin{array}{c}\because {\rm{l}}_{\rm{FG}}={\rm{l}}_{\rm{CD}}=m=\frac{\rm{rcos\varphi }}{1+\rm{sin\varphi }} \\ \therefore {\rm{l}}_{\rm{GI}}={\rm{l}}_{\rm{FI}}-{\rm{l}}_{\rm{FG}}=r-\frac{\rm{rcos\varphi }}{1+\rm{sin\alpha }}\end{array}$$

Therefore, $\Delta =\rm{r}-\frac{\rm{rcos\varphi }}{1+\rm{sin\varphi }}$. The meaning of Δ value: As can be seen from the above analysis, if the distance between the upper and lower steel rails in the simplified model of the steel rail and the rail wheel is s, in the actual structure of the steel rail and the rail wheel, the distance is equivalent to an increase of 2∆ on the original basis, that is, the total distance is s+2∆. Thus,the rail biting condition 1 in the actual structure of the steel rail and the rail wheel can be obtained as: l_GI > s+2∆

Therefore, the value of Δ is represented as follows $\Delta =r-\frac{r\mathrm{cos}\varphi }{1+\mathrm{sin}\varphi }$. The interpretation of Δ: As can be seen from the above analysis, if the spacing between the upper and lower steel rails in the simplified models of the steel rail and steel rail wheel is denoted by $s$, then in the actual structures of the steel rail and steel rail wheel, the spacing is equivalent to being increased by 2Δ on the original basis, making the total spacing $s+2\Delta$. Consequently, the first rail biting condition for the actual structures of the steel rail and steel rail wheel can be derived as:

$$\sqrt{{\left(s-2\delta \right)}^{2}+{t}^{2}}>s+2\Delta$$

It’s obtained:

9 $$\begin{array}{c}\frac{s-\sqrt{{\left(s+2\Delta \right)}^{2}-{D}^{2}+{d}^{2}}}{2}> \delta \end{array}$$

structures of the steel rail and steel rail wheel can be derived as biting condition 2 is that the Angle of deviation α when rail biting occurs during operation is greater than the critical deviation Angle [α], that is, α > [α]. The calculation formula for the critical deviation Angle is as follows:

The second rail biting condition for the actual structures of the steel rail and steel rail wheel is: When rail biting occurs during scraper operation, the skew angle $\alpha$ must be greater than the critical skew angle $[\alpha ]$, i.e., $\alpha> [\alpha ]$, and the formula for calculating the critical skew angle is as follows:

10 $$\begin{array}{c}\left[\alpha \right]=arccos\frac{s+2\Delta -2\delta }{\sqrt{{\left(s-2\delta \right)}^{2}+{D}^{2}-{d}^{2}}}-arccos\frac{s+2\Delta }{\sqrt{{\left(s-2\delta \right)}^{2}+{D}^{2}-{d}^{2}}}\end{array}$$

## Analysis of the causes of derailment and the conditions of rail biting

### Causes of derailment

From the rail biting analysis mentioned above, we can see that if the gap δ between the inner side of the steel rail wheel flange and the steel rail side meets the formula (9), the rail biting will occur when the driving deflection reaches a certain angle; If the gap between the inner side of the steel rail wheel flange and the steel rail side does not meet the formula (9), the rail biting will not occur. At this time, as the difference in the walking distance of the upper and lower steel rail wheels becomes larger, the driving deflection angle also becomes larger. When the deflection angle reaches the state shown in Figure 8(b) in the diagram of normal operation and derailment of the driving steel rail wheel, the upper and lower steel rail wheels have completely left the steel rail, and the phenomenon of derailment will occur.

### Analysis of derailment conditions for simplified rail wheel model

According to formula (5), when $\delta \ge \frac{s-\sqrt{{s}^{2}-\left({D}^{2}+{d}^{2}\right)}}{2}$, rail biting will not occur, but derailment may still happen.

As shown in Figure 8(a), the normal and derailment conditions of the wheel on the steel rail, the length of line segment UV is:

11 $$\begin{array}{c}{l}_{\mathrm{UV}}=s-2\delta +2x\end{array}$$

In the formula, s represents the distance between the inner sides of the upper and lower steel rails; δ represents the clearance between the inner side of the steel wheel flange and the side of the steel rail; x represents the distance from the outer end face of the steel rail wheel to the inner side of the flange;

As shown in Figure 8(b), when the scraper is deflected and points U and V are located inside the upper and lower steel rails, derailment will occur. At this time, the length ${l}_{\mathrm{UK}}$ of projection line segment UK of line segment UV in the vertical direction is no more than s.

In $\Delta \mathrm{UVK}$, according to the trigonometric function relationship, we can get:

12 $$\begin{array}{c}\mathrm{cos}\gamma =\frac{{l}_{\mathrm{UK}}}{{l}_{\mathrm{UV}}}=\frac{s}{s-2\delta +2x}\end{array}$$

From this, we can get the derailment condition of the scraper:

13 $$\begin{array}{c}\gamma \ge arccos\frac{s}{s-2\delta +2x}\end{array}$$

In the formula, γ is the deflection angle of the scraper.

### Analysis of derailment conditions based on the actual structure of steel rails and wheels

In the actual structure of steel rails and wheels, due to the presence of chamfers on the steel wheels, this factor needs to be taken into account in the calculation of derailment conditions. Figure 9 is a simplified schematic diagram of the chamfer of the steel wheel involved in the derailment situation.

As can be seen from Figure 9, the length of line segment UV in the actual structure of the steel wheel is:

14 $$\begin{array}{c}{l}_{\mathrm{UV}}=s-2\delta +2x-2{c}_{1}\end{array}$$

In the formula, ${c}_{1}$ represents the dimension of the chamfer on both sides of the steel wheel’s end face.

By substituting Equation (14) into Equation (12), we can obtain the critical angle for derailment in the actual structure of the steel wheel:

15 $$\begin{array}{c}\mathrm{cos}\gamma =\frac{{l}_{\mathrm{UK}}}{{l}_{\mathrm{UV}}}=\frac{s}{s-2\delta +2x-2{c}_{1}}\end{array}$$

Based on this, the actual derailment condition for the steel wheel during operation is:

16 $$\begin{array}{c}\gamma \ge arccos\frac{s}{s-2\delta +2x-2{c}_{1}}\end{array}$$

## Experimental analysis and verification

**Fig. 10:**
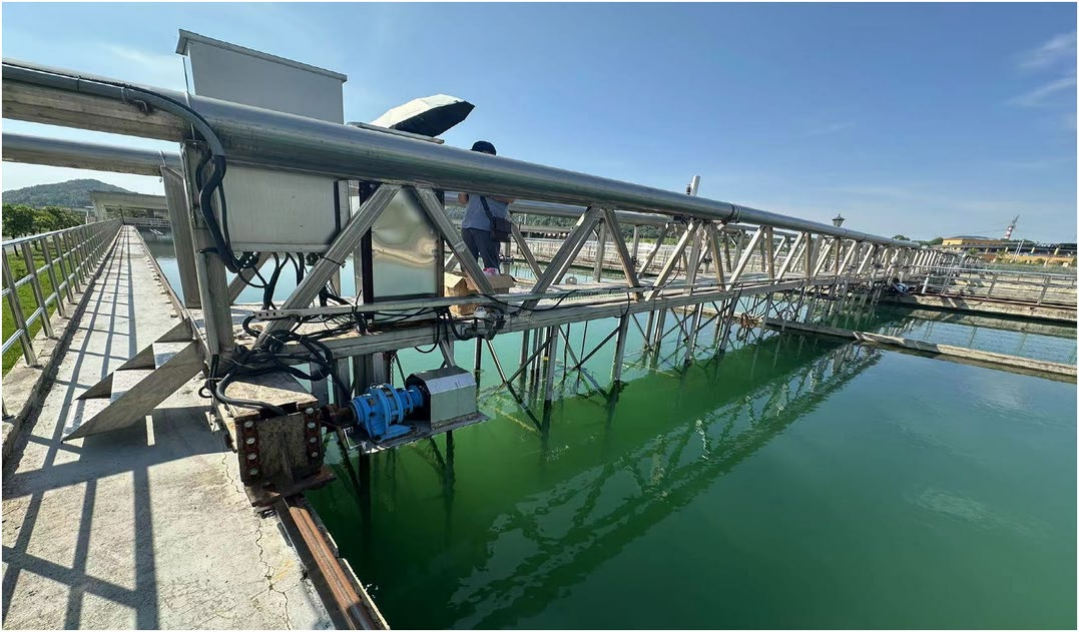
On-site photo of the sludge scraper.

Regarding the field experiment on the traveling error of large-span sludge scraper, relevant experimental equipment was set up in the Jinkou Water Plant in Wuhan, as shown in Figure 10. The traveling error of large-span scraper directly affects the operational stability and safety of the equipment, especially under complex environmental conditions such as rainfall, which may exacerbate the rail biting phenomenon. The purpose of this experiment is to comprehensively monitor the distance changes between the wheels and the rails as well as external environmental factors through precision sensor technology, so as to more accurately assess the impact of these factors on rail biting errors and provide data support for subsequent rail maintenance and design.

**Fig. 11:**
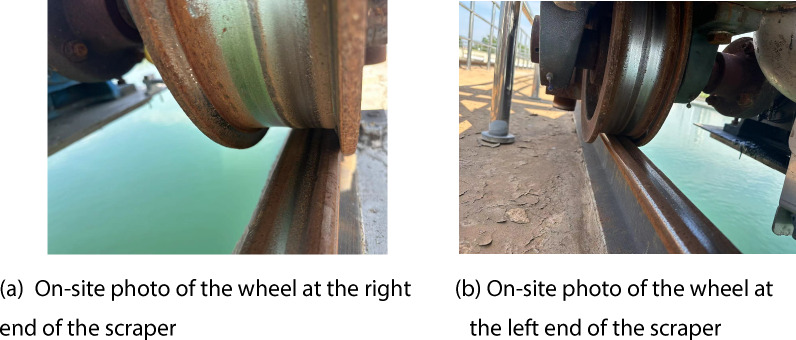
Examples of the wheels at the bottom of the scraper (**a**) On-site photo of the wheel at the right (**b**) On-site photo of the wheel at end of the scraper the left end of the scraper.

**Fig. 12:**
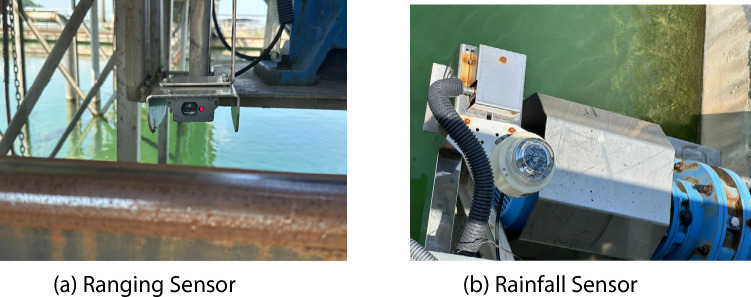
Examples of sensors (**a**) Ranging sensor (**b**) Rainfall sensor.

There are two wheels at each end of this large-span scraper, totaling four wheels as shown in Figure 11. A ranging sensor is installed next to each wheel, as shown in Figure 12(a), to precisely measure the distance variations between the wheel and the rail. This high-precision laser ranging sensor boasts a measurement accuracy of ±0.1 millimeters, effectively capturing subtle distance changes of the wheel during operation, making it suitable for complex industrial environments. In addition, a rainfall sensor is also equipped on the sludge scraper, as shown in Figure 12(b), to detect precipitation conditions in the environment. The intelligent rain gauge from Controls Co. is chosen for its high sensitivity and automatic calibration function, allowing for accurate recording of real-time rainfall. The study investigates whether the degree of rail slipperiness affects the rail biting error under different rainfall conditions.

**Fig. 13:**
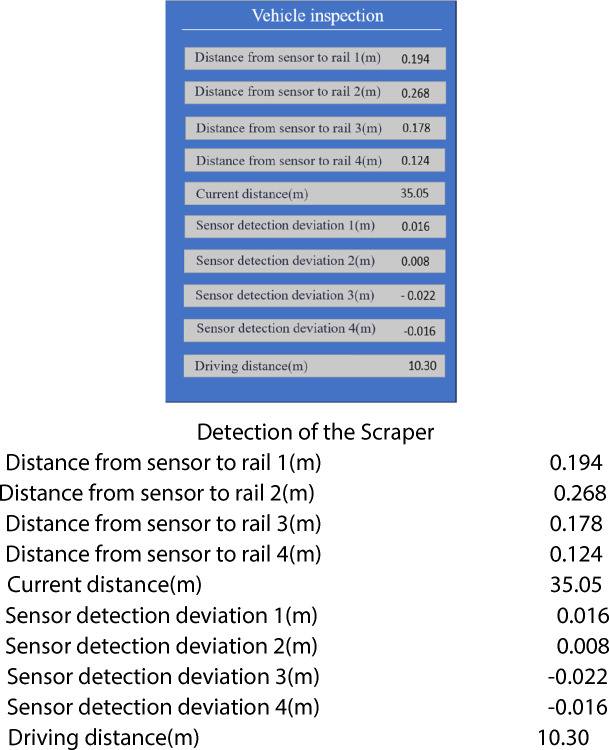
Sensor monitoring data chart.

**Fig. 14:**
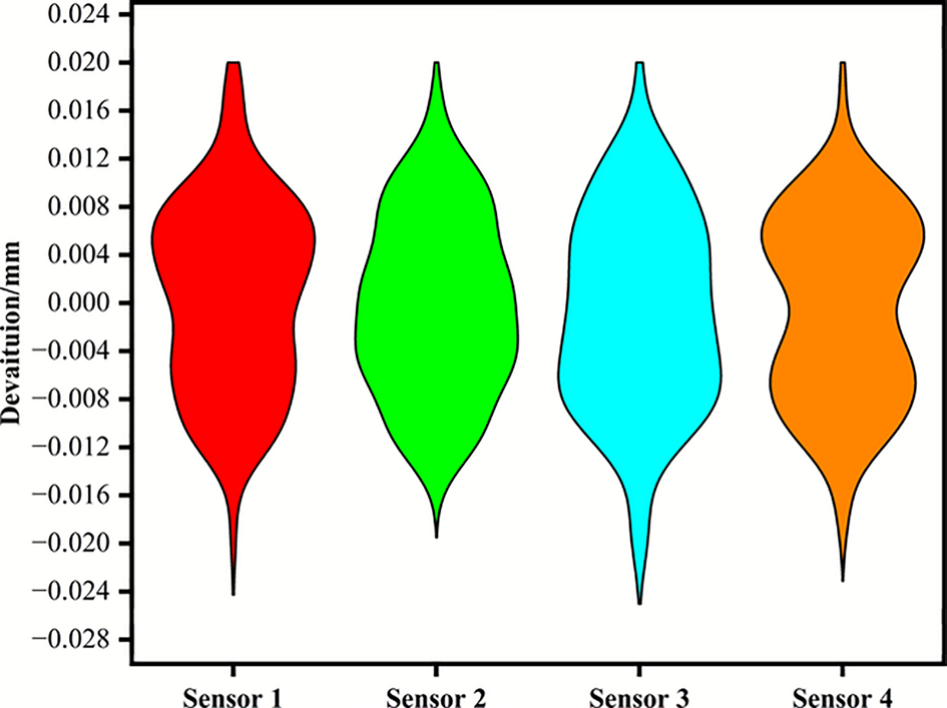
Probability distribution diagrams of measurement errors for different sensors.

Figure 13 shows the real-time data monitored by the ranging sensors during the traveling of the scraper, using the currently measured data as an example. The scraper travels from the calibrated starting point to the current position, with an experimental displacement of 10.30 meters. At the current coordinate, the ranging sensors measure the distances from the total of four wheels (two at each end) to the rail, which are 0.194m, 0.268m, 0.178m, and 0.124m respectively. Each sensor has a set standard value for the distance to the rail, which varies due to differences in installation positions. Therefore, as long as the distance between the sensor and the standard value falls within the allowable range, the wheel is considered centered. As shown in the figure, the differences between the four sensors and the standard distances are 0.016m, 0.008m, −0.022m, and −0.016m, all within the ±0.02m range. When the measured difference exceeds the set difference, it is determined that rail biting has occurred, and the controller issues speed control instructions to the generator to perform correction operations, ensuring that the gap between the inner side of the rail wheel flange and the side of the steel rail remains within the normal range. During the experiment, the current error values of the four sensors were each recorded 100 times. Meanwhile, the critical angles were calculated both without Δ correction and with Δ correction based on the different values measured by the four sensors. Then, the collected data were statistically analyzed, and the results are shown in Figure 14 and Table 1 respectively. The results indicate that after introducing Δ correction, the equipment operates stably and the prediction error is reduced by approximately 91.025%%. This verifies the practicality of the method.

**Fig. 15:**
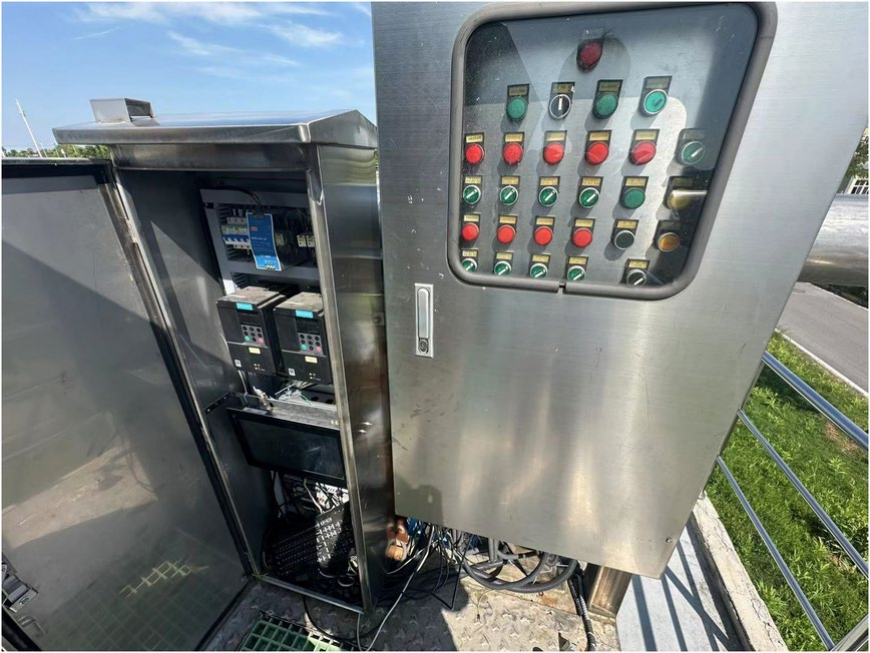
Operation control cabinet of the Scraper.

Moreover, a control cabinet is equipped on the observation platform as shown in Figure 15. The data collected by the sensors is uploaded to the control platform for data analysis and processing to achieve real-time monitoring purposes. The sensors sample multiple times per second, uploading data to the central control platform in real-time. The PLC system within the control cabinet is responsible for preliminary processing of sensor data, which is then transmitted to the remote server via industrial Ethernet. More complex analysis is conducted on the server. The real-time monitoring platform is also equipped with data visualization functions, which can dynamically display the wheel distance variation trends and rainfall data, providing decision support for on-site operators.

## Summary

Based on a review of domestic and international research on the causes, analysis, and improvement measures of rail biting phenomena, this paper theoretically analyzes the formation reasons and critical conditions of rail biting and derailing during the operation of large-span scraper. It identifies the conditions and calculation formulas for rail biting and derailing, proposes allowable error ranges for the gap between the inner side of the rail wheel flange and the side of the steel rail as well as the deviation angle of rail biting, and compares them with actual conditions. Taking into account the influence of errors such as chamfers and transition arcs, the theoretical analysis is refined, providing a theoretical basis for future synchronized control of large-span scraper. Based on the theoretical analysis of errors, an experimental platform has been set up outdoors to measure the difference in wheel-rail distances between normal operation and the critical conditions of rail biting and derailing in real-time, further verifying the authenticity and reliability of the theory.

## Data Availability

The datasets used and analyzed during the current study are available from the corresponding author on reasonable request.
